# The Anatomy of the Excito-Motor System

**Published:** 1847-02

**Authors:** 


*The Anatomy of the Excito-Motor System.* By MARSHALL HALL.—The history of the protracted disputes on this topic would be full of instruction, but it is not my present intention to write it. My object is, to lay before the readers of the Lancet, in a few words, (I am always afraid of occupying their time and my own needlessness,) *the* argument, or rather, *the* plain and simple PROOF, of the distinct anatomy of the excito-motor system.

Does any one doubt the distinct anatomy of the system of cerebral nerves—of the nerves of sensation and volition?

The *very same* proof which exists of this part of the nervous system, exists in regard to the excito-motor system. It exists in the pneumogastric nerve, or, as it may be better designated, the pneumogastric system of nerves.

If the pneumogastric be sentient at all, it is the least sentient of all incident nerves. What is it then? It is excito-motor! It is, emphatically, the internal, excito-motor nerve!

Why go to complex structures, when a simple one exists? Why go to the lower order of animals when the mammalia, and even the human being, afford us the proof we require?

The superior and the inferior laryngeals are the associated excitor and motor nerves of the larynx.

The bronchials are associated excitor and motor nerves of the bronchia.

The pharyngeals and œsophageals are associated excitor and motor nerves of the pharynx, of the œsophagus, and of the cardia.

Lastly, the most strikingly, the pulmonic part of the pneumogastric nerve is, as the associate of the diaphragmatic and intercostals, the internal excitor of respiration.

There is, in short, as I have said, the *same* proof the distinctness of the excito-motor system of nerves, as of the sentient and voluntary, and it is both idle and ridiculous to dispute the fact any longer, or to appeal to other parts of the nervous system than the grand pneumogastric, or to other tribes of animals than the mammalia, for proofs not needed. As confirmations of a truth already established, these researches are, of course, interesing enough. I am myself preparing a paper on the pneumogastric system of animals of limited and diffused respiration in the mammalia; and in birds and insects. In birds, the spinal nerves are, doubtless, in their distribution to the diffused breathing cells, analogous to the pulmonic branches of the pneumogastric. In insects, *each segment* with its spiracles (analogues of the larynx, trachea, and bronchia,) is endowed with a nervous system entirely analogous to the laryngeals, and to the pulmoniac branches of the pneumogastric, and the diaphragmatic, or intercostals! Then we have to inquire into the nature and office of the lateral nerve in fishes. As in birds, the respiratory nerves are, probably, equally used for flight and for respiration; so in fishes, the lateral nerve is, probably, for swimming and for respiration.

But to return to my topic. The proof of the distinct anatomy of the excito-motor system is afforded by the pneumogastric—the internal, purely, or almost purely, excito-motor nerve.

If, however, we would examine other and more complicated tissues, the proof lies, not, I fear, in the dissection and tracing of fibres, but in physiological experiments; the cerebral system is so tested, *in*-excitor throughout—in its centre, in the nerves of special sense; the excito-motor system is, in its centre, and in its incident and reflex relations, what its designation implies.

It is pitiable that there should any longer be any dispute on the subject, or that detraction should still attempt to wrest the credit of adducing *the* proof, in any degree, from myself, or from physiology.

Amongst other attempts of this kind, one has been to propose a change in the designation which I had given to the nerves of the reflex are—and a most unfortunate change, too. The incident and reflex *imply* some very definite association, or LAW-*relation*, between the two—a real phenomenon of the most remarkable kind. But the terms *afferent* and *efferent*, are, in this respect, utterly insignificant; whilst the meaning which these words do convey, of something borne to and fro, is probably altogether erroneous.

The ray of light, which is now incident and immediately afterwards reflected, is the *same* ray, modified, directed, and returned by the reflector, whether it consist in locomotive particles, or in vibration. The same idea is attempted to be conveyed by the terms incident and reflex nerve. There is, in these nerves, and in their connection through the spinal marrow, some extraordinary recondite connection, so that, for example, the excitation of the superior laryngeal sends forth some mysterious messenger to the medulla-oblongata, whilst this returns it in the just channel, the inferior laryngeal, so as to effect the closure of the larynx; whilst the excitation of the pulmonic branches of the pneumogastric excites, through the diaphragmatic and intercostal nerves, the contraction of the muscles of inspiration, precisely, definitely, and no other.

The ordinary reflexion of a ray of light, or the polarisation of a ray of light, is not more definite.

The effect produced is obviously *designed*, not by the animal—for its brain may be removed without interfering with this process—but by an omniprovident Creator. This obvious design has misled many to think that there are feeling and volition in the spinal marrow.

The terms incident and reflex are therefore full of meaning; whilst the terms afferent and efferent either convey no meaning at all, an erroneous one. In this suggestion, the Law *of association* of the effects of excitement, its incident course, its modification and direction by the spinal marrow, its reflex course and destination, were unperceived.

How much, then, is conveyed or implied in that one word, *reflex*, —incidence, reflexion, appropriate combination, and destination! And how devoid of all meaning are the words afferent, and efferent not very modestly attempted to be substituted for it!

I beg my reader to study and compare the physiological movements in the acts of inspiration, with their pathological forms in asphyxia: the first are reflex, normal, and beautifully appropriate; the second are, in respect, abnormal and deranged.

My opponents are much disposed to speak of the class of reflex actions, in general terms, as known to Ridi, Whytt, &c. This is another ill-chosen but deceptive phrase. The reflex *actions* as I have *always* said, were spoken of by many previous physiologists; but the phrase I have adopted from the very beginning,—for the very title of my first paper,—was reflex *function;* and this expression, with its fulness of meaning, as applied to all the acts of ingestion and egestion in the animal economy, had been used, could have been used by no one; for as the idea of an incident excitor nerve, with its physiological relations, did not exist in anatomy, so the idea of a reflex function, with its anatomical relations, did not exist in physiology.—*Lancet.*

